# A Pilot Study of Integration of Medical and Dental Care in 6 States

**DOI:** 10.5888/pcd18.210027

**Published:** 2021-07-22

**Authors:** Molly Linabarger, Monique Brown, Nita Patel

**Affiliations:** 1 Centers for Disease Control and Prevention, Division of Oral Health, Atlanta, Georgia in 2018–2020; 2 Centers for Disease Control and Prevention, Division of Oral Health, Atlanta, Georgia

## Abstract

**Introduction:**

Poor oral health affects overall health. Chronic diseases and related risk factors such as tobacco use or consuming sugar-sweetened beverages can also increase a person’s risk of periodontitis. Given the linkages between oral health and certain chronic diseases, we conducted a pilot study to facilitate intradepartmental collaborations between state chronic disease and oral health programs.

**Methods:**

State health departments in 6 states (Alaska, Colorado, Georgia, Maryland, Minnesota, and New York) collaborated to develop and implement projects that addressed oral health and the following chronic diseases or risk factors: obesity, diabetes, heart disease, stroke, and tobacco use. States developed various projects, including media campaigns, clinical education, and screening and referrals. We used a mixed-methods approach to understand barriers to and facilitators of states’ increasing collaboration and implementation of pilot projects. In-depth interviews were conducted with 12 staff (1 from oral health and 1 from chronic disease for each state). We also reviewed state-submitted documents and performance measures.

**Results:**

All 6 states increased collaboration between their oral health and chronic disease programs and successfully implemented pilot projects. Collaboration was facilitated by investing in relationships, championing medical–dental integration, and meeting and communicating frequently. Barriers to collaboration included the perception of oral health in chronic disease programs as separate and distinct from other chronic diseases and the structure of funding. The pilot projects were facilitated by partner support, providing technical assistance to clinics, and working early on referral networks. Barriers to implementing the pilot projects included gaining clinician buy-in and establishing referral networks.

**Conclusion:**

This pilot study demonstrated that by fostering collaboration, state health departments are able to train dental and medical clinicians, deliver clinical preventive education to patients, implement referral systems, and deliver impressions via media campaigns.

**Summary**

**What is already known on this topic?**

Poor oral health is linked to chronic diseases such as diabetes, cardiovascular disease, and obesity.

**What is added by this report?**

Six state health departments conducted 2-year pilot projects to promote collaboration between their oral health and chronic disease programs. States were able to increase collaboration, train oral health and medical professionals, deliver clinical preventive education to patients, implement referral systems, and deliver education via media campaigns.

**What are the implications for public health practice?**

Collaborations between oral health and chronic disease programs can result in promising projects that address common risk factors for oral health and chronic disease.

## Introduction

Poor oral health, which includes dental caries (tooth decay), periodontal disease (gum disease), and oral cancer, affects quality of life for millions of Americans (1,2). Tooth decay is one of the most common chronic diseases in the United States. About 1 in 4 US children aged 2 to 5 years, 52% of children aged 6 to 8 years, 90% of adults aged 20 to 64 years, and 96% of adults aged 65 or older experience dental caries (3). Approximately 42% of adults aged 30 or older had periodontal disease in 2009–2014 (4). In 2016, nearly 45,000 new cases of cancer of the oral cavity and pharynx were diagnosed in the United States, and more than 10,000 people died from those diseases (5).

Studies show that poor oral health is linked to chronic diseases such as diabetes, cardiovascular disease, and obesity (6,7). Emerging research indicates a possible a 2-way relationship between diabetes and periodontitis. Research suggests that diabetes, especially when poorly controlled, is a risk factor for periodontitis, and conversely, people with diabetes may be at increased risk of periodontitis (7). Although causality has not been established, studies suggest associations between periodontitis and cardiovascular disease (8) and between periodontitis and obesity (9). Research suggests that obesity could be a potential risk factor for periodontal disease, especially among younger people (10). The linkage between poor oral health and overall health also includes risk behaviors such as tobacco use (11) and consuming foods and beverages with high levels of added sugar (12).

Few public health programs in the United States integrate oral health and chronic disease programs. Although oral disease and chronic disease are linked, dental and medical health care systems are not. The Institute of Medicine and others have proposed integrating oral health into the medical health care system to promote better health and improve access to both dental and medical preventive services (13–15). Some agencies have also called to examine the role of medical–dental integration in reducing oral health disparities (16) and to increase oral health equity (17).

Given that research shows links between oral health and certain chronic diseases (6–12) and a lack of integration of oral health and chronic disease programs within state health departments, the Centers for Disease Control and Prevention (CDC) initiated a pilot project, Models of Collaboration, in 2016 in which it funded 6 state health departments to promote collaboration between programs addressing oral health and chronic disease (eg, diabetes, heart disease) or risk behaviors (eg, smoking, high-sugar diet).

## Purpose and Objectives

The purpose of Models of Collaboration was for the chronic disease and oral health programs of state health departments to collaboratively develop and implement 2-year chronic disease prevention pilot projects that integrated activities from both chronic disease and oral health programs. The aim of the state pilot projects was to facilitate, strengthen, and increase collaboration between oral health and chronic disease programs at the state health department around common risk factors for oral health and chronic disease; build synergy; and maximize resources to improve oral health and decrease associated comorbid chronic diseases. We provided guidance to states to select strategies that focused on the prevention of selected chronic diseases or risk behaviors of mutual importance to both the states’ oral health and chronic disease programs, such as obesity prevention; diabetes, heart disease and stroke prevention; or tobacco control. As described earlier, these are risk factors known to increase the risk of periodontitis and poor oral health.

## Intervention Approach

We provided a general framework to states as they developed their pilot projects. This framework included 1) convening an advisory panel of key chronic disease and oral health personnel to oversee the 2-year project; 2) creating and refining a project work plan that used oral health program activities to have an impact on 1 chronic disease; 3) using oral health program staff, partners, and activities to implement the project work plan; 4) assessing the project though process and outcome evaluation measures; 5) building communication among state chronic disease and oral health program staff to strengthen collaboration between the programs; and 6) reporting project outcomes to state and national chronic disease and oral health partners.

The rationale for providing a framework was to identify commonly used program components that states would implement as part of the project to maintain structure and a level of consistency in the development of pilots across the 6 states, given that each state would be selecting its own prevention program for oral health and chronic disease. For example, the development and participation of advisory panels would reflect an intent by the leadership of both the chronic disease and oral health programs to jointly commit time and resources to the pilot projects. A work plan is a useful project implementation tool in that it defines strategies, activities, evaluation and performance measures, and project timelines and assigns tasks to staff of both the oral health and chronic disease programs. Building communication activities included sharing project work plans and progress with chronic disease and oral health partners and oral health staff participating in development of the state’s chronic disease prevention plan. Finally, we recommended that states disseminate project outcomes and evaluation findings to state and national chronic disease and oral health partners to monitor program outcomes, build an evidence base for program interventions, and drive continuous program improvement.

States developed interventions on the basis of a combination of contextual factors including existing relationship between state oral health and chronic disease programs, state priorities, and recommendations from an advisory panel of key chronic disease and oral health personnel. All state interventions selected were evidence-based: consumption of foods and beverages with high levels of sugar in relation to dental caries (12), screening for periodontitis among people with diabetes (7), poor oral health among people who use tobacco (11), and associations between periodontitis and cardiovascular disease (8).

Six state health departments (Alaska, Colorado, Georgia, Maryland, Minnesota, New York) developed state-specific pilot projects, which were conducted from August 2016 through August 2018, 5 of which were clinical interventions (eg, diabetes risk assessments and testing in federally qualified health centers [FQHCs], administering periodontal self-assessments in community health clinics). In addition to key project outcomes listed, all pilot states had success stories, including some life-saving referrals (Table 1).

**Table 1:** Project Success Stories, Pilot Study of Medical–Dental Collaboration in 6 US States, 2016

State	Success Story
Alaska	**Desire for materials.** One of the key successes of Alaska’s project was the interest generated for the project-created communication materials. After the communications guide was published, several groups, including the Special Supplemental Nutrition Program for Women, Infants, and Children (WIC), Supplemental Nutrition Assistance Program (SNAP), and 2 tribal organizations, invited staff to present and train on the guide. Having a variety of state agencies ask that their staff have access to and be trained on the communication materials showed how successful the project had become. **Perception of practitioners.** Another key success of Alaska’s project was the positive perception of project-created communication materials among practitioners. After presenting at the Alaska Native Tribal Health Consortium, project staff learned that some clinicians were already familiar with and using the materials in their practices. The program received a great deal of positive feedback, including the impact these resources were having in their communities. Clinicians indicated their desire to have the project implemented in Head Start programs and schools. The passion that the training and materials evoked from clinicians was inspiring. The development and publication of the guide started conversation among different agencies and clinicians on difficult issues.

Colorado	**Self-esteem.** A patient came to the Colorado Coalition for the Homeless, an FQHC that provides various services to homeless people, including medical and dental care, to receive dental care to improve his chances at getting a job. He had not seen a medical doctor in years, and while there, he agreed to take the verbal risk assessment for diabetes. When his score came up high, the clinician did a point-of-care hemoglobin A1c (HbA1c) screening. The test showed that the patient probably had diabetes. Through the Diabetes Oral Health Integration project, the patient was referred to primary care for diagnosis, education, medications, and other needs for care. Because of the screening and subsequent care, this patient was able to improve the appearance of his teeth, felt ready to get a job, and was connected with medical clinicians to help control his diabetes. **Access to integrated care.** A patient visited the Colorado Coalition for the Homeless dental clinic for a problem-focused visit. They had previously been told by clinicians to monitor their HbA1c levels because of a family history of diabetes; however, because of limited access to care, the patient had not been screened in many years. Before the Diabetes Oral Health Integration project, testing a patient’s HbA1c levels would not be included in dental care. Luckily for this patient, under the project protocols, the dental clinician referred the patient to a medical clinician on site for more testing that same day. The client was grateful to be able to receive both dental and medical care at the same visit. Another patient who came in for dental care received a point-of-care HbA1c screening and was surprised to learn that they had elevated glucose levels. The patient was referred to the medical clinic for a same-day appointment where they were diagnosed, given diabetes education, and prescribed appropriate medications. Without the Diabetes Oral Health Integration project, this patient would not have been screened, diagnosed, or treated for their diabetes, and their oral and overall health would have continued to suffer.

Georgia	**Impact on clinicians**. One key success from Georgia’s Models of Collaboration project was its impact on dental clinicians. During clinician training, staff were able to use interactive presentation software to gain real-time insights from participants. They conducted pre- and post-training session surveys to understand how the presentation affected clinicians. After the training session, clinicians were more likely to report interest in seeing pregnant clients, accepting Medicaid for pregnant clients, and educating patients on tobacco cessation. This confidential expression of increased interest showed program staff that clinicians were excited about what the staff had to say. Having real-time survey data where all participants could see the results also served as a motivating factor to the other clinicians in the room who saw that their peers were interested in changing their practices toward serving pregnant women and providing smoking cessation counseling.

Maryland	**Life-saving care.** A patient served by Maryland’s Models of Collaboration project credited the program with saving his life. That patient came to a dental clinic for a comprehensive oral exam and full mouth x-rays. He was not exhibiting any symptoms and did not report pain or feeling ill. Still, as part of the new intake protocol, the chairside assistant took the patient’s blood pressure and found it to be high (147/101). After taking the blood pressure a second time to confirm, the patient was referred to his primary care clinician and urged to seek care as soon as possible because he had no previous history of hypertension. The patient was so concerned upon learning this that he instead went directly to a nearby emergency department (ER). At the ER, he passed out and his heart stopped several times. Thanks to the screening provided by Maryland’s Models of Collaboration project, this patient was quickly diagnosed with heart failure and received the necessary care. The importance of hypertension screening from a dental clinician was underlined by Maryland’s statewide media campaign, “2 minutes with your dentist can save your life.”

Minnesota	**Establishment of referral network.** A key success of Minnesota’s pilot programs was the establishment of a referral network among private practices. One dental clinic, in particular, was extremely dedicated to creating a reliable medical referral pathway for patients who were identified as needing medical attention. The lead dentist at this clinic identified the ideal urgent care walk-in clinic to receive patients on the basis of its proximity to the dental clinic. Establishing communication with the clinic proved challenging, but the lead dentist persisted. Ultimately, he succeeded in making contact, outlined the project, and demonstrated to senior leaders of the urgent care center the need for a formalized relationship between these 2 facilities. Soon a workflow between the 2 clinics was established. Reflecting back, staff from the dental clinic credit the “pressure” of receiving funding to establish a project for pushing them to be persistent enough to establish a relationship.

New York	**Formative research.** As part of the Models of Collaboration pilot project, the New York State Oral Health Program worked with their partners in the Adolescent Health Program, who already had established partnerships with adolescent health and after school programs, to conduct formative research on attitudes toward sugar-sweetened beverages. This partnership gave the oral health program access to their target audience — young people — to conduct focus groups to improve their messages, and some of the results were surprising. For example, one strategy they thought would be effective in communicating with young people, using celebrities or athletes, was identified by focus group participants as not appealing. Without this vital feedback, the program may have developed products and disseminated them in ways that did not connect with their target audience. As a result, by avoiding traditional strategies such using celebrities, they hope their materials will also stay relevant longer. The relationship with the Adolescent Health Program allowed project staff to quickly reach their target audience and learn valuable insights that they believe resulted in a stronger, more sustainable media campaign. **Variety of dissemination methods.** Although they had originally planned to do only a social media campaign, New York State was able to disseminate their message on a much larger scale. As they were working on the social media campaign, they collaborated with their contracted advertising agency to reallocate funds to add out-of-home advertising to the media campaign. This redistribution of funds allowed them to develop a variety of out-of-home advertisings, including posters, billboards, interior bus signs, exteriors of bus shelters, and cooler clings and “one sheets” in convenience stores. In some cases, the Drink Water messages were placed alongside the competing soft drink advertisements on coolers in convenience stores. A close partnership with schools and chronic disease prevention partner organizations facilitated the dissemination of their posters, allowing messages to be displayed to students in classrooms, cafeterias, clinic waiting rooms, gyms, and more. By closely collaborating with their advertising agency, redistributing their funds, and disseminating products through partners, New York State was able to greatly increase the number of people who saw their important messages.

**Sugar-sweetened beverages.** Alaska developed a clinical intervention in community dental practices and tribal health organizations to reduce the consumption of sugary drinks and encourage drinking (preferably fluoridated) water through a pilot in which dental clinicians incorporated a counseling intervention to address sugary drink consumption among parents and children. Key project outcomes included training 125 participants and developing and distributing more than 600 communication guides to be used by oral health professionals as structured training materials to discuss sugar-sweetened beverages with patients.

New York developed and implemented a multimedia marketing campaign among African American and Hispanic adolescent males in western New York to decrease sugar-sweetened beverage consumption and encourage drinking (preferably fluoridated) water. Key project outcomes included a media campaign that delivered more than 25 million impressions (ie, the number of times a piece of media content such as a billboard or social media message is consumed).

**Tobacco cessation.** Georgia piloted a tobacco cessation project for dental clinicians working with pregnant women aged 18 to 24 who were eligible for Medicaid and WIC (Special Supplemental Nutrition Program for Women, Infants, and Children), provided tobacco cessation and quitline referral training for dental clinicians serving this population, developed a tobacco prevention tool kit for oral health clinicians, and created a media campaign. Key project outcomes included training 62 oral health clinicians, and 14,780 tobacco quitline caller referrals made by dental professionals.

**Hypertension and tobacco cessation.** Maryland developed and implemented hypertension and tobacco use screening and referral in dental practices and created a social marketing campaign to promote hypertension screenings in the dental setting among African American women aged 35 to 65 at risk for hypertension. Key project outcomes included screening 36,996 patients and referring 2,689 to primary care. The pilot also implemented a media campaign that delivered more than 3 million impressions.

**Hypertension and periodontal disease**. Minnesota developed and implemented a program with bi-directional referrals in community health clinics for periodontitis and hypertension. Key project outcomes included dental offices referring 3,646 patients to clinicians and medical offices administering 844 periodontal self-assessments.

**Diabetes and periodontal disease.** Colorado collaborated with an FQHC to facilitate training, screening, and bi-directional referral for periodontitis and diabetes/prediabetes. Key project outcomes included delivering 461 diabetes risk assessments and 100 prediabetes (hemoglobin A1c) tests.

## Evaluation Methods

At the end of the 2 years, we evaluated Models of Collaboration with 2 objectives: 1) to determine facilitators and barriers for collaboration between state oral health and chronic disease health programs, and 2) to determine barriers and facilitators in the development and implementation of pilot projects. We used a mixed-methods evaluation study design, collecting both qualitative and quantitative data. The primary data collection tools were in-depth interviews, a review of state-submitted documents, and performance measures.

### Data sources

**In-depth interviews.** We conducted in-depth interviews to better understand project implementation, facilitators, barriers, and lessons learned. Two in-depth interviews (1 interview with staff from the oral health program and a second interview with staff from the collaborating chronic disease program) were conducted with state health department staff from each of the 6 project states for a total of 12 in-depth interviews. Staff were purposively selected for their experience with the pilot project. Interviews were conducted via Skype or telephone from January 11 through April 19, 2019, by M.L., a trained and experienced qualitative interviewer. Verbal consent was obtained from all interview participants. Interviews ranged from 47 minutes to 1 hour 45 minutes (average = 1 hr 17 min) and were recorded on Skype for Business recording software and a digital recorder. CDC reviewed this study for human subjects protection and deemed it to be nonresearch.

**State-submitted documents.** As part of Models of Collaboration, states were required to submit yearly performance measure and narrative progress updates and a final evaluation report. Two yearly reports were submitted, 1 for each year of the project, which included quantitative data collected by the states to measure progress on their self-established performance measures and a narrative with the following elements:

- Dissemination of evaluation results
- Enhancements made based on evaluation findings
- Successes
- Challenges
- CDC program support to awardees

We reviewed these state-submitted documents to identify project facilitators and barriers and to create project-specific probes for in-depth interviews. Twenty-eight documents in total were analyzed.

**Performance measures.** States developed performance measures based on key outputs or outcomes (Table 2). Each state was required to set targets and to collect and submit data for these performance measures each year. For performance measures, the 6 states developed a numeric indicator value and identified whether they had met the target, were in progress to meet the target or the work was ongoing, or had not met the target.

**Table 2:** Performance Measures, Pilot Study of Medical–Dental Collaboration in 6 US States, 2016

Short-Term Outcomes	Intermediate Outcomes
Established a pilot project that integrated oral health and chronic disease program staff and resources. Increased awareness of importance of oral health in chronic disease conditions among state health department staff Increased communication and information sharing between chronic disease and oral health programs Incorporated oral disease control systems and concepts into the state’s chronic disease work plans Improved messaging about the importance of oral health in chronic disease programs	Developed public health programs that used oral health infrastructure to affect chronic disease performance measures. State chronic disease program staff collaborated with oral health program staff and partners. Used oral health professionals in chronic disease prevention programs.

### Data analysis

We transcribed interviews verbatim, developed a codebook based on the interview guide, and iteratively updated the codebook throughout the coding process. Themes based on the identified barriers and facilitators were developed by comparing responses across and between states. For state-submitted documents, we coded narrative portions of these documents and analyzed in the same manner as in-depth interviews. In-depth interviews and narrative portions of state-submitted documents were analyzed by using ATLAS.ti (ATLAS.ti Scientific Software Development).

We reviewed state-submitted performance measure updates. Because states developed their own performance measures for different pilot projects, performance measures varied by state. For example, for the outcome “increased incorporation of oral disease systems and concepts into the state’s chronic disease work plans,” 1 state defined this performance measure as the number of instances of incorporation of oral disease systems and concepts into the state’s chronic disease work plans, whereas another state defined it as the number of strategic plans developed where oral health program staff and their partners were engaged. Additionally, not all states conducted pilot projects on the same chronic disease prevention program or risk factor (ie, some states worked on diabetes prevention and others worked on smoking cessation). States developed indicators and target values solely on the chronic disease or risk factor they selected, creating wide variability in target values for indicators among the 6 pilot states. Because of these differences, performance measure data were used to determine key successes for each state and whether state-determined targets were met, but these data were not compared across the pilot projects.

## Results

All 6 states successfully implemented the general framework provided (ie, advisory panel, work plan, implementing work plan, assessing project, building communication, and reporting project outcomes) as they collaborated with their respective oral health and chronic disease health programs. We report key facilitators and barriers to state health department collaboration and pilot project implementation synthesized across all 6 states.

### Collaboration

#### Facilitators

Collaboration between state chronic disease program staff, oral health program staff, and their partners increased in all 6 states. All were successful in convening and collaborating with an advisory panel made up of internal and external oral health and chronic disease personnel. States increased integration of oral health and chronic disease by adding elements of oral health to state chronic disease work plans and vice versa, creating communication materials that addressed both oral health and chronic disease and increasing the frequency of communication between programs. Key facilitators to improving collaboration at the state health department included 1) investing in relationships, 2) championing medical–dental integration, and 3) meeting and communicating frequently.

State representatives identified building and maintaining relationships between members of the oral health and chronic disease programs as a facilitator of several different aspects of collaboration. Relationships helped in the identification and recruitment of advisory panel members. As a result of the relationships built, oral health representatives were invited to participate in other aspects of chronic disease programming. Building strong relationships was also key to helping states continue to collaborate despite frequent staff transitions, an issue faced by several states.

Championing medical–dental integration was another facilitator of collaboration at the state health department. Interest in medical–dental integration helped program staff actively look for opportunities to collaborate. Because funding was provided to only 1 program in each of the state health departments (to the chronic disease program or the oral health program), staff of the other program were not always funded to work on the project. Believing in the idea of the project and seeing the benefit for both programs helped facilitate collaboration, even in cases where funding was not provided by Models of Collaboration.

Meeting and communicating frequently helped facilitate collaboration. Frequent meetings helped ensure that staff continued to collaborate despite staff transition. In addition, meetings helped build and maintain relationships, facilitate information sharing, and solidify the collaboration between the programs. Overall, states reported that meeting frequency varied between weekly and monthly and was either in person or by telephone.

#### Barriers

The most common barrier to improving collaboration between programs was that oral health was viewed as separate and distinct from other chronic diseases, which affected the states’ ability to collaborate on work plans and communication materials. One oral health staff member found it difficult to integrate work plans because they perceived oral health as being interactive earlier in life than other chronic diseases and at more points throughout the lifespan (ie, oral health programming can target young children, pregnant women, adults, and the elderly, whereas programming for other chronic diseases mostly targets adults). In response to developing shared communication materials, a chronic disease staff member from another state said, “It comes across as dental oral health is kind of a standalone, whereas other chronic diseases like cardiovascular and diabetes are more connected at the hip.” Respondents reported that this disconnect between oral health and other chronic diseases had less of an impact for those working with chronic diseases and associated risk factors that had stronger, more widely accepted evidence of a causal relationship with oral health (ie, consumption of sugar-sweetened beverages, smoking) than for those with less evidence of a causal relationship (ie, hypertension, diabetes).

Funding for Models of Collaboration was provided to 1 program in the state health department. Other program staff were not always funded or fully funded. Even among staff who were interested in medical–dental integration, a lack of direct funding made it difficult for them to dedicate time to the project. Staff reported feeling overwhelmed by their workload and found it difficult to spend much time on projects they were not funded to develop or implement.

### Pilot projects

Overall, all 6 states, regardless of the type of chronic disease or risk factor they worked with, were able to develop and implement pilot projects. Key outcomes of these pilot projects included training of oral health and medical clinicians, delivering clinical preventive education to patients, implementing referral systems, and delivering impressions through media campaigns (Table 1).

#### Facilitators

Key facilitators to developing and implementing the pilot projects were partner support, providing technical assistance to clinics, and working on referral networks early. As part of the project guidelines, states were asked to convene an advisory panel to support the development and implementation of their pilot project. The advisory panel provided key clinical expertise to state health department staff. This clinical expertise included developing clinical workflows, providing guidance on referral systems, clinical guidelines, billing, and reviewing communication materials for clinical accuracy.

Several states found that providing technical assistance to the clinics implementing the medical–dental collaboration program improved implementation. As expected in a pilot project, clinics faced issues when incorporating screening and referral processes into their established workflows. By communicating with the implementing clinics, state health departments were able to learn about problems that the clinics were facing and collaborate with their advisory panels to develop solutions to these problems. In addition, maintaining strong relationships with the clinics allowed state health departments to share information and lessons learned across different clinics. This support to the clinics amounted to clinical quality improvement practices.

When patients were screened and identified as being at high risk for a chronic disease, referral protocols needed to be in place. Several states that struggled to create referral networks between clinics provided a few strategies to facilitate this process. For example, states recommended working on building referral networks early in the process, even before the official start of a project. Building strong relationships early on with potential referring clinics through consistent communication can facilitate the establishment of referral networks.

#### Barriers

Key barriers to developing and implementing the pilot projects were gaining clinician buy-in and developing and implementing referral networks. Several states said that getting clinician buy-in to the project at the clinic level was difficult. State health department staff faced resistance when they asked clinicians to change their workflow to incorporate screening, referrals, or education. Clinicians told state health department staff that because their time with patients was already limited, adding an additional task such as screening, referrals, or education was difficult. States responded by working with clinics to establish workflows that accommodated these additional tasks.

Some clinics experienced difficulties in creating referral networks. State health department staff found that establishing these networks took longer than anticipated. One dental clinic was able to establish a referral procedure only after multiple attempts at contacting the medical clinic, highlighting the importance of persistence. Some barriers to establishing referral networks were a lack of medical practices near the dental clinic and an inability of potential medical and dental referral sites to take on new patients, especially those without health or dental insurance. Clinics also struggled to track and measure referral completion, partially because medical and dental clinic health records were not interoperable.

## Implications for Public Health

Although the mouth is part of the body, oral health has historically been treated as separate from medical health. This distinction dates back to the origins of dentistry as a profession (18) and the lack of inclusion of dentistry during the establishment of medical schools in the United States, still evident in the mostly separate care system that we have today (19). The long-standing perception that oral health is separate and distinct from overall medical health was cited as a barrier to collaboration by the 6 state health departments that manifested in challenges in implementing pilot projects, especially in integrating work plans and developing communication materials. Low prioritization of oral health on the political agenda is another barrier to integrating oral health into primary care (20).

Several reviews of medical–dental integration found that having strong leadership champion the integration facilitates the process, in part through educating public health professionals and clinicians about the importance of oral health (20,21). Relationship-building was key to increasing collaboration. For some states, educating the collaborating program at the state health department was an important aspect of building relationships as was creating and engaging champions for medical–dental integration from their partner program. Models of Collaboration allowed for the growth of relationships among pre-existing champions who previously had not been able to collaborate on medical–dental integration because they lacked explicit funding. Partnerships and common vision were a facilitator in other medical–dental integration projects (20).

States that implemented pilots in clinical settings faced a unique set of challenges. The biggest of these challenges was developing referral networks between clinics where none previously existed. This included getting clinician buy-in at the clinic level, changing workflow to incorporate screenings, referrals, and education. Studies have shown that although clinician opinions of using dental settings to screen for chronic diseases were generally positive, some barriers — including workload, time, cost, and patient willingness — remained (22). Our 6 states found that gaining clinician buy-in was difficult in some cases, requiring that clinics work on their own or in collaboration with the state health department to overcome issues related to workload and time (developing workflows that work for each clinic) and patient willingness (developing a standard explanation of the purpose of screening). Establishing and implementing these processes took time and persistence.

After screening patients, clinicians referred those at risk to appropriate clinicians for care. As with other projects (21), just as medical clinicians had difficulty finding dentists to accept patients, dental clinicians had difficulty finding nearby medical clinicians who would accept patients, especially patients without insurance. Health record incompatibility was also identified as a barrier to integration (20,21). The inability of dental records to “talk to” electronic health records makes the referral process difficult. All 5 states implementing clinical interventions reported difficulties related to health record incompatibility.

Quality improvement is important in implementing medical–dental integration projects (21). Providing support to clinics and sharing lessons learned across clinics was an important way the states improved integration. The Models of Collaboration pilot projects addressed some of the issues identified by an environmental scan of public health medical–dental integration efforts (23). One issue was a lack of established protocols for implementing integrated activities. Five of 6 projects turned to local experts through an advisory panel to develop guidance for clinics. The environmental scan also recommended that projects prioritize local community needs through formative research, which several states, including New York, did (Table 1).

Sustainability of medical–dental integration remains an issue (19,20) and has been identified by several reviews (20,21,23). Sustainability of funding at the clinic level, specifically sustainability of integrated practices after grant funding was completed, was a concern among health department staff. State health departments funded only 1 of the 2 programs (oral health or chronic disease), so members of the partner program found it difficult to collaborate in a sustainable manner. CDC funded this pilot project for 2 years but has since expanded funding for future medical–dental integration projects for longer periods. Lessons learned from this pilot project were used to improve the new long-term CDC project (24).

Because of our study’s small sample size, our findings are not generalizable; however ,they can provide lessons for future medical–dental integration projects. Our 6 states were funded for a short time — 2 years. A few states reported that more than half of that time was spent developing relationships between oral health programs and chronic disease programs, leaving little time to implement the pilot projects themselves, especially those in clinic settings. This short time frame limited the ability of states to collect clinical outcome data, corroborated by an environmental scan, which found very limited outcome data (23). Finally, a few states faced frequent staff turnover. This affected continuity of project planning and implementation and affected the ability of some interviewees to respond to select interview questions.

Evidence is slowly emerging on the effectiveness of integration models (25), with some evidence pointing to the need for reform of the oral health care system (26) and a recognition of all social determinants of health connecting oral health and overall medical health, especially during the COVID-19 pandemic (27). State health departments are uniquely positioned to support medical–dental integration. Our pilot study showed that through collaboration, state oral and chronic disease programs can leverage funding to provide training and increase screenings and referrals for oral diseases that share risk factors with chronic diseases. Additional studies are needed to further understand some of the logistical challenges in implementing integration projects, including building effective and sustainable referral networks.
